# Tape strip expression profiling of juvenile dermatomyositis skin reveals mitochondrial dysfunction contributing to disease endotype

**DOI:** 10.1172/jci.insight.179875

**Published:** 2025-03-13

**Authors:** Jessica L. Turnier, Sarah M.H. Vandenbergen, Madison E. McClune, Christine Goudsmit, Sophia Matossian, Meredith Riebschleger, Nadine Saad, Jacqueline A. Madison, Smriti Mohan, Johann E. Gudjonsson, Lam C. Tsoi, Celine C. Berthier, J. Michelle Kahlenberg

**Affiliations:** 1 Division of Pediatric Rheumatology, Department of Pediatrics;; 2 Division of Rheumatology, Department of Internal Medicine;; 3 Department of Dermatology;; 4 Department of Computational Medicine and Bioinformatics;; 5 Department of Biostatistics; and; 6 Division of Nephrology, Department of Internal Medicine, University of Michigan, Ann Arbor, Michigan, USA.

**Keywords:** Autoimmunity, Dermatology, Inflammation, Autoimmune diseases, Clinical practice, Expression profiling

## Abstract

Skin inflammation in juvenile dermatomyositis (JDM) can signal disease onset or flare, and the persistence of cutaneous disease can prevent complete disease remission. The noninvasive study of cutaneous expression signatures through tape stripping (TS) holds the potential to reveal mechanisms underlying disease heterogeneity and organ-specific inflammation. The objectives of this study were to (a) define TS expression signatures in lesional and nonlesional JDM skin, (b) analyze TS signatures to identify JDM disease endotypes, and (c) compare TS and blood signatures. Although JDM lesional skin demonstrated interferon signaling as the top upregulated pathway, nonlesional skin uniquely highlighted pathways involved in metabolism, angiogenesis, and calcium signaling. Both lesional and nonlesional skin shared inflammasome pathway dysregulation. Using unsupervised clustering of skin expression data, we identified a treatment-refractory JDM subgroup distinguished by upregulation of genes associated with mitochondrial dysfunction. The treatment-refractory JDM subgroup also demonstrated higher interferon, angiogenesis, and innate immune expression scores in skin and blood, though scores were more pronounced in skin as compared with blood. TS expression signatures in JDM provided insight into disease mechanisms and molecular subgroups. Skin, as compared with blood, transcriptional profiles served as more sensitive markers to classify disease subgroups and identify candidate treatment targets.

## Abstract 2

We identified expression signatures in lesional and non-lesional skin of juvenile dermatomyositis patients that highlight novel biological pathways for understanding disease heterogeneity and targeting treatment.

## Introduction

Juvenile dermatomyositis (JDM) is a multisystem inflammatory disease of childhood that predominantly affects skin and muscle. Cutaneous inflammation is frequently the first recognized symptom at disease onset or flare and assists in diagnosis (1). Skin disease commonly persists even in the absence of clinically active muscle disease and is often more refractory to treatment (2, 3). Cutaneous damage is common and can be debilitating, leading to scarring or atrophy in up to 40%, calcinosis in 20%, and lipodystrophy in 13% of children (4). Moreover, persistent skin disease in JDM has been demonstrated to predict a longer time to disease remission and a chronic disease course (5, 6). Skin, as opposed to muscle involvement, is associated with nailfold capillary loss (7) and premature cardiovascular disease (8), suggesting that skin disease may be a more reflective biomarker of vasculopathy and subclinical inflammation in JDM and should be a larger focus of overall disease activity assessment.

Although the majority of studies to date have focused on characterization of peripheral blood transcriptomes to understand disease pathophysiology (9, 10), tissue-specific expression signatures hold the potential to be more informative of organ-specific disease mechanisms and treatment targets than blood (11, 12). Skin-specific expression signatures may also lend insight into individual disease heterogeneity and define disease endotypes to inform precision medicine approaches to care in JDM. Whereas skin biopsies are invasive and infrequently obtained in routine clinical practice, tape stripping is a noninvasive, painless, established method to sample skin using adhesive stickers and has been utilized diagnostically in multiple other skin diseases (13–15). In the pediatric population, tape stripping in children with atopic dermatitis has been paired with transcriptome profiling to define novel disease endotypes (13) and has highlighted key genes and pathways in disease pathogenesis (15, 16). Gene expression signatures obtained via tape stripping from both nonlesional and lesional skin of patients with cutaneous lupus erythematosus (CLE) have been shown to distinguish patients with CLE from healthy controls, and interferon signatures from tape stripping were noted to be more pronounced as compared with peripheral blood (17).

Although tape stripping is traditionally considered to sample the epidermis, it is possible to obtain expression signatures from immune cells (13, 18, 19). Tape stripping methodology, as compared with biopsy, may also be used to sample nonlesional skin and reveal subtle preclinical changes ahead of diagnosis or disease flare. It is recognized that even nonlesional JDM skin is altered at both a histopathologic and transcriptional level and has been shown to contain increased numbers of myeloid cells and dysregulation of innate immune pathways, despite absence of clinical inflammation (20, 21). Tape stripping signatures from children and adults with atopic dermatitis and psoriasis have also demonstrated nonlesional skin to be altered from healthy controls (14, 16). Subtle preclinical changes can be effectively detected using tape stripping, which may provide a more robust assessment of expression signatures originating from keratinocytes in the upper layers of the epidermis (22).

It is becoming increasingly recognized that immune cells are shaped and educated through intercellular communication networks and the inflammatory milieu unique to their tissue microenvironment. In fact, the cutaneous microenvironment has been demonstrated to modulate inflammatory phenotypes of myeloid cells in adult cutaneous lupus (23). Although there have been recent advances in characterizing immune cell subpopulations present in lesional JDM and DM skin using imaging mass cytometry (24, 25), further studies are needed to understand the mechanistic role of identified immune cell subpopulations in disease pathogenesis and to delineate the role of stromal cell populations in the immune response.

We thus performed a pilot study to establish the feasibility of obtaining gene expression signatures from adhesive sampling of skin in pediatric autoimmune disease, specifically for patients with JDM. The primary objectives of this study were to (a) define cutaneous gene expression signatures in lesional and nonlesional JDM skin recovered with tape stripping as compared with healthy controls to delineate biological mechanisms critical to JDM cutaneous inflammation, (b) compare tape stripping signatures with previous microarray data from full-thickness JDM skin biopsies, (c) determine the ability of tape stripping expression data to delineate JDM disease subgroups, and (d) compare cutaneous and peripheral blood expression signatures collected at the same time points to identify organ-specific signatures.

## Results

### JDM cohort clinical characteristics at enrollment visit.

There were 28 JDM and 20 healthy control (CTL) patients in this study. All patients had sampling of nonlesional (NL) skin. Within our JDM cohort, lesional (L) skin was also sampled if rash was present. In the JDM cohort, 17/28 (60.7%) had both a L and NL skin sample collected, and 11/28 (39.3%) had only a NL skin sample collected (Table 1, Table 2, and Supplemental Table 1; supplemental material available online with this article; https://doi.org/10.1172/jci.insight.179875DS1). The mean age for JDM diagnosis was 9.0 years for patients with both L and NL skin samples and 8.2 years for JDM patients with NL skin samples only. At the time of tape stripping, the mean age for JDM patients with both L and NL skin sampled (13.3 years) did not differ significantly from JDM patients with NL skin sampling only (11.0 years). Our JDM cohort was predominantly female (23/28, 82.1%) and White (20/28, 71.4%).

We had 2 treatment-naive patients in our cohort, both in the group of patients with L and NL skin samples collected. There was no significant difference in disease duration between the 2 patient groups (4.3 years for JDM with L and NL skin sampling and 2.7 years for NL sampling only). There were more patients with skin-predominant disease in the NL only relative to the group of patients having both L and NL skin samples (5/11 or 45.4% relative to 1/17 or 5.8%). Our JDM cohort had overall low skin disease activity scores. Cutaneous Dermatomyositis Disease Area and Severity Index (CDASI) activity scores were higher in patients having both L and NL skin sampling, with a mean Cutaneous Dermatomyositis Disease Area and Severity Index activity score of 7.1 in the L and NL skin group relative to 1.5 in the NL skin only group. Muscle disease activity scores, as assessed by Manual Muscle Testing (MMT-8) and Childhood Myositis Assessment Scale (CMAS), were similar in both cohorts. Physician’s global assessment of disease activity (PGA) scores were slightly higher in the group of patients having both L and NL skin samples (3.2 relative to 1.8). There was not a significant difference in testing positive for presence of an MSA between the L and NL skin group and the NL skin only group (8/15 or 53.3% relative to 3/10 or 30.0%), and TIF1γ was the most represented MSA in the cohort (4/15 or 26.7%). Of note, 2/17 patients in the L and NL skin group and 1/11 patients in the NL skin only group had an unknown MSA status and were not included in these percentages.

### Transcriptome analysis of JDM lesional skin highlights interferon and immune activation signatures.

**Figure 1:**
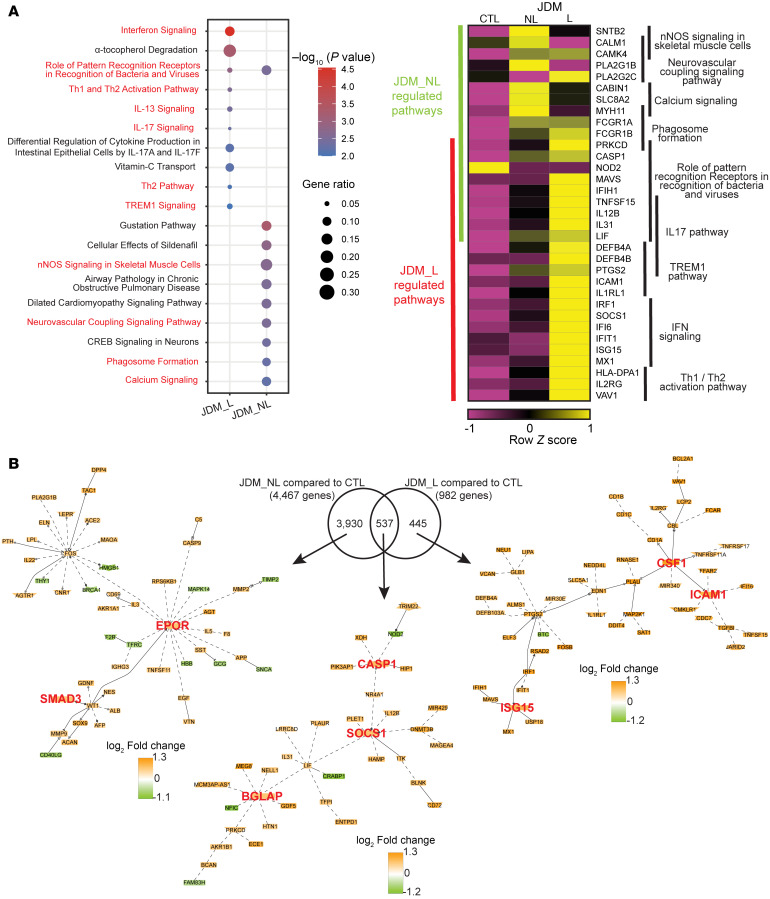
Biological signatures identified in JDM L and NL skin compared with CTL. (**A**) The left panel displays the top 10 biological pathways (*P* value < 0.05) regulated in JDM L (*n* = 17) and NL skin (*n* = 28) compared with CTL skin (*n* = 20); the right panel displays selected genes from relevant pathways. Canonical pathway *P* values were computed by the Ingenuity Pathway Analysis (IPA; QIAGEN) software. nNOS, neuronal nitric oxide synthase. (**B**) Literature-based network analysis of shared and unique genes in JDM L and NL compared with control skin.

To account for patients who had multiple samples from the same tissue type (different time points, NL and/or L), we first combined all samples of a specific tissue type from each patient before differential expression analysis (Supplemental Figure 1). Upon comparison of all JDM L relative to CTL skin samples, we identified 982 differentially expressed genes (DEGs), including 929 upregulated and 53 downregulated genes (FDR < 0.10; Supplemental Table 2). Interferon signaling was the top upregulated pathway (*P* value < 0.0001), with the top 10 upregulated pathways also including role of pattern recognition receptors in recognition of bacteria and viruses (*P* value = 0.0014), Th1 and Th2 activation (*P* = 0.0028), IL13 signaling (*P* = 0.0036), IL17 signaling (*P* = 0.0048), and TREM1 signaling (*P* = 0.01) (Figure 1A, Supplemental Figure 2, and Supplemental Table 3). Central nodes in literature-based network analysis for lesional JDM skin included *ISG15*, *ICAM1*, and *CSF1*, as well as genes involved in the interferon response, immune cell trafficking, and monocyte and macrophage survival (Figure 1B). The top predicted upstream regulators in lesional skin included *IRF1* (IPA *Z* score = 3.95, *P* = 2.07 × 10^–7^) and *SOCS1* (IPA *Z* score = –3.37, *P* = 1.57 × 10^–9^), which are both genes involved in the regulation of IFN signaling (Supplemental Table 4).

### Transcriptome analysis of JDM NL skin is distinct from L skin and exhibits predominant upregulation of metabolic signaling pathways.

Interestingly, JDM NL skin demonstrated a higher number of DEGs (*n* = 4,467, FDR < 0.10) in relation to CTL skin than was seen in L skin comparisons. JDM NL skin displayed 4,138 upregulated and 329 downregulated genes compared with CTL (Figure 1B and Supplemental Table 2). Pathway analysis demonstrated a striking difference compared with JDM lesional skin, with regulation of genes involved in nNOS signaling in skeletal muscle cells (*P* value = 0.002), neurovascular coupling signaling (*P* = 0.003), phagosome formation (*P* = 0.005), and calcium signaling (*P* = 0.006) (Figure 1A, Supplemental Figure 2, and Supplemental Table 3). In NL skin, *EPOR*, which encodes the erythropoietin receptor, and *SMAD3*, which functions in TGFB1 signaling, were among the nodes identified on network analysis (Figure 1B). The top predicted activated upstream regulators expressed in NL skin were the transcription factors *HNF1A* (*Z* score = 4.4, *P* value = 0.0139), which is involved in regulation of glucose metabolism, and *ARNT2* (*Z* score = 4.3, *P* value = 0.0361), which can participate in regulation of transcriptional response to hypoxia (Supplemental Table 4).

### JDM L and NL skin demonstrate shared activation of innate immune signaling pathways.

Direct comparison of JDM L with NL skin demonstrated only 10 DEGs (FDR < 0.10), all downregulated in JDM L skin, indicating that the transcriptome of JDM NL skin appears more similar to JDM L skin rather than CTL skin (Supplemental Table 2). Interestingly, 2 of these genes were *LOR* and *FLG2*, both related to epidermal barrier function and cornified envelope formation, which have been previously described as downregulated in systemic lupus erythematosus L skin (26). There were 537 DEGs in JDM L overlapping with the NL skin when compared with CTL (Supplemental Table 2 and Figure 1B), and top upregulated pathways in this gene set in common between L and NL skin included role of pattern recognition receptors (*P* = 0.0026), inflammasome pathway (*P* = 0.0129), IL13 signaling (*P* = 0.0191), and IL17 signaling (*P* = 0.0245) (Supplemental Table 3). Literature-based network analysis of those 537 overlapping DEGs in JDM L and NL skin compared with CTL highlighted increased mRNA expression of *BGLAP*, encoding osteocalcin, a bone matrix protein potentially involved in calcinosis, as well as *CASP1*, which can activate IL1B and IL18 in the proper context (Figure 1B). We also identified *SOCS1*, which is involved in negative regulation of cytokines. The heatmap displayed in Figure 1A and Supplemental Figure 2 highlights some of the upregulated genes in key pathways unique to each JDM L and NL skin and in common between JDM L and NL skin relative to CTL.

### Tape stripping and full-thickness skin biopsies share a common interferon expression signature in L skin and alteration in cellular and metabolic signaling pathways in NL skin.

**Figure 2:**
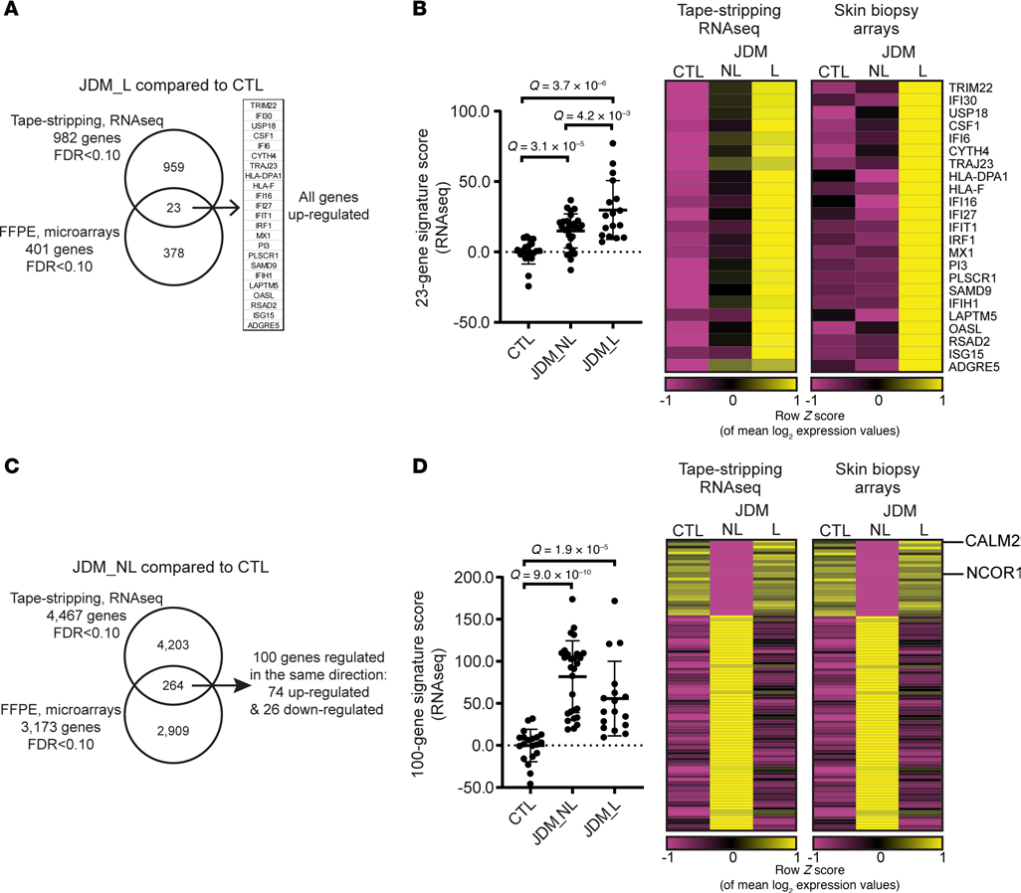
Comparison of tape stripping with full-thickness skin biopsy expression signatures. (**A**) JDM lesional skin. FDR was extracted from the Limma analysis (**B**) A 23-gene signature from overlap genes in L expression datasets. Data are presented as mean ± SD. Unpaired 2-tailed parametric Student’s *t* test with Benjamini-Hochberg correction was used for the comparison between 2 groups. (**C**) JDM NL skin. FDR was extracted from the Limma analysis. (**D**) A 100-gene signature from overlap genes in NL expression datasets. Data are presented as mean ± SD. Unpaired 2-tailed parametric Student’s *t* test with Benjamini-Hochberg correction was used for the comparison between 2 groups. RNA-Seq: *n* = 20 CTL, *n* = 17 JDM_L, *n* = 28 JDM_NL; microarrays: *n* = 8 CTL, *n* = 9 JDM_L, *n* = 6 JDM_NL.

To understand the extent to which expression signatures acquired via tape strips reflect the whole skin tissue transcriptome, we compared the JDM tape stripping RNA-Seq transcriptional profile with the full-thickness FFPE skin biopsy microarray expression profile that we previously published (27). The JDM L tape stripping RNA-Seq and full-thickness skin biopsy microarray expression data comparison showed a prominent interferon-stimulated gene expression signature, verifying that tape stripping can effectively recover signatures reflecting the immune dysregulation occurring in inflamed skin. There were a total of 23 genes commonly upregulated in expression datasets from both lesional tape stripping and skin biopsy samples (Figure 2A), including *TRIM22*, *IFI30*, *USP18*, *CSF1*, *IFI6*, *CYTH4*, *TRAJ23*, *HLA-DPA1*, *HLA-F*, *IFI16*, *IFI27*, *IFIT1*, *IRF1*, *MX1*, *PI3*, *PLSCR1*, *SAMD9*, *IFIH1*, *LAPTM5*, *OASL*, *RSAD2*, *ISG15*, and *ADGRE5*. A 23-gene signature score generated using these 23 genes showed that this signature was also higher in JDM NL skin tape stripping samples, indicating the detectable presence of an interferon signature even in JDM NL skin but at a lower level than seen in L skin (Figure 2B and Supplemental Figure 3). When comparing JDM NL skin datasets, we noted 100 common DEGs between tape stripping and skin biopsies, with 74 upregulated and 26 downregulated genes (Supplemental Table 2; Figure 2, C and D; and Supplemental Figure 3). Calcium signaling, nNOS signaling in skeletal muscle cells, and mitochondrial biogenesis were among the top represented pathways (*P* value < 0.05; Supplemental Table 3). Of note, *CALM2* and *NCOR1* from the mitochondrial biogenesis pathway were both downregulated in JDM NL relative to CTL skin, indicating possible dysregulation in mitochondrial homeostasis in JDM skin even in the absence of clinical inflammation.

### Unsupervised hierarchical clustering identifies a JDM molecular subgroup with an expression signature characterized by mitochondrial dysfunction.

**Figure 3:**
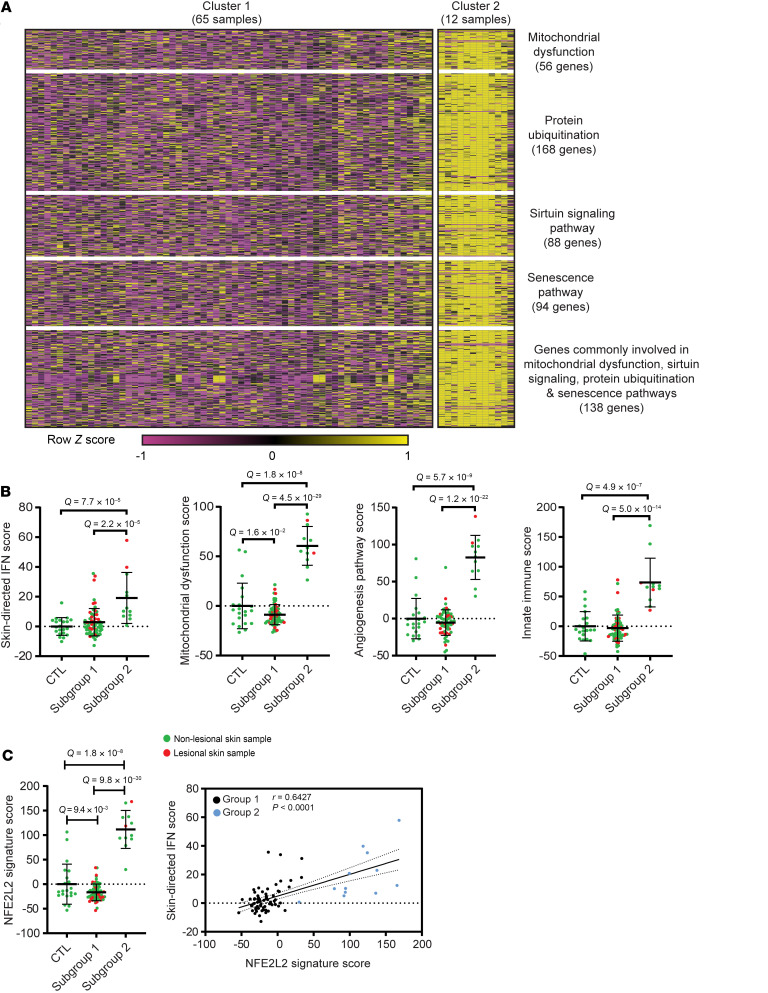
Unsupervised hierarchical clustering of skin expression data identifies subgroups of patients with JDM. (**A**) Heatmap of selected DEGs representing pathways reflecting 2 JDM skin subgroups. (**B**) JDM subgroup 2 demonstrated higher skin-directed interferon, mitochondrial dysfunction, angiogenesis, and innate immune expression scores in skin compared with subgroup 1 and healthy controls. (**C**) The *NFE2L2* signature score was higher in subgroup 2 compared with subgroup 1 and positively associated with the skin-directed interferon score. Unpaired 2-tailed parametric Student’s *t* test with Benjamini-Hochberg correction was used for the comparison between 2 groups. Cluster 1: *n* = 65 total samples (*n* = 45 JDM_NL and 20 JDM_L) corresponding to *n* = 28 patients; cluster 2: *n* = 12 total samples (*n* = 10 JDM_NL and 2 JDM_L) corresponding to *n* = 8 patients.

Unsupervised hierarchical clustering from all the CTL and JDM skin samples identified 2 JDM subgroups with distinct tape stripping expression profiles (corresponding to 65 samples or 28 patients in cluster 1 and 12 samples or 8 patients in cluster 2) (Supplemental Figure 4, A and B). Clustering of enrollment tape stripping samples only (1 sample/individual) produced similar results (Supplemental Figure 4C). We therefore included all tape stripping samples per individual in the analysis. The 6,773 DEGs (FDR < 0.01, absolute log_2_ fold-change ≥ 1.0; Supplemental Table 2) distinguishing the 2 subgroups represented pathways involving mitochondrial dysfunction, sirtuin signaling, oxidative phosphorylation, protein ubiquitination, and senescence (*P* value < 0.0001) (Figure 3A and Supplemental Table 3). JDM subgroup 2 demonstrated higher skin-directed interferon, mitochondrial dysfunction, angiogenesis, and innate immune expression scores in skin compared with JDM subgroup 1 and healthy CTL patients (Figure 3B). Upon comparing clinical disease characteristics between subgroups, the 2 subgroups did not separate by L/NL skin status or disease duration or activity (Table 3, Supplemental Table 5, and Supplemental Figure 4). However, patients represented in subgroup 2 were more likely to still be on steroids after similar disease duration, indicating a higher frequency of treatment-refractory disease (Table 4 and Supplemental Table 5), suggesting that tape stripping expression signatures may hold potential to aid in clinical stratification of JDM patients with a different disease subtype that could be more refractory to standard treatment. Of note, the 2 patients in our cohort with treatment-naive samples were split between subgroups, supporting that our findings are not entirely driven by treatment effect. Pathway-based expression scores in skin also did not associate with steroid dose (Supplemental Figure 5).

### NFE2L2 is the top upstream regulator in the JDM treatment-refractory molecular subgroup.

*NFE2L2* (log_2_ fold-change = 1.26, FDR < 0.0001), a transcription factor involved in cytoprotective response to oxidative stress and innate immune signaling, was the top upstream regulator activated in JDM subgroup 2 (IPA *Z* score = 10.74, *P* value = 6.1 × 10^–14^) (Supplemental Table 4). There were 221 genes downstream of *NFE2L2*, from which 72 were involved in at least 1 of the following pathways: mitochondrial dysfunction, protein ubiquitination, sirtuin signaling, unfolded protein response, myelination signaling, and autophagy (in the top 15 pathways from the 6,773 genes regulated in JDM subgroup 2 compared with subgroup 1, Supplemental Table 3 and Supplemental Table 6). A total of 71 of the 72 genes were upregulated in subgroup 2 compared with subgroup 1 and were used to generate an *NFE2L2* signature score (Supplemental Table 6). The defined *NFE2L2* signature score was higher in subgroup 2 and positively associated with the skin-directed interferon score (*P* = 0.6427, *P* < 0.0001) (Figure 3C), indicating an association of *NFE2L2* and interferon in JDM pathophysiology.

### Validation of JDM molecular subgroup differentiated by mitochondrial dysfunction using independent JDM cohort microarray expression dataset from full-thickness skin biopsies.

**Figure 4:**
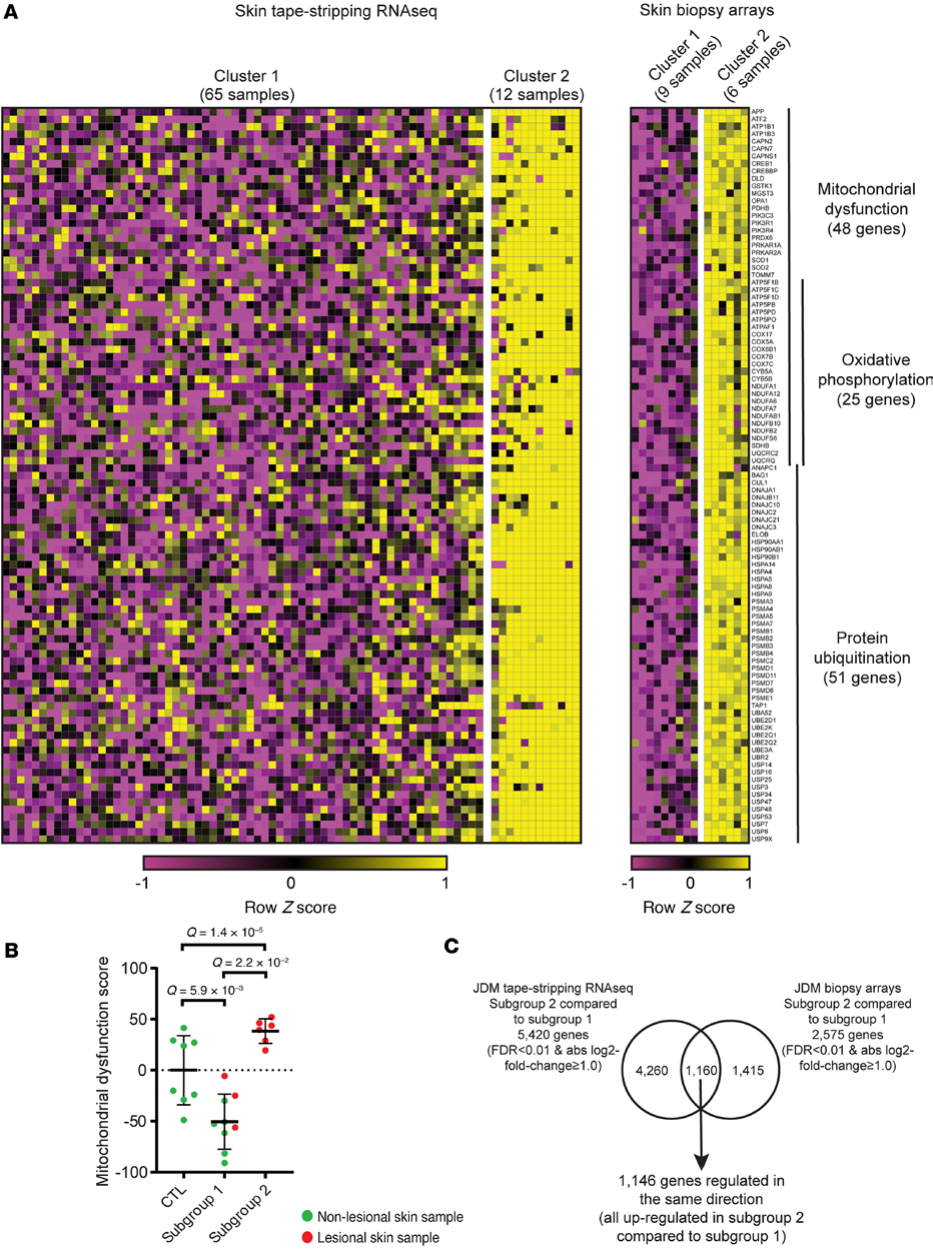
Validation of a JDM subgroup expression signature using a JDM skin biopsy microarray dataset. (**A**) Heatmap of the DEGs representing selected pathways commonly activated between the 2 identified JDM subgroups in both skin tape stripping and biopsy. (**B**) Subgroup 2 demonstrated higher mitochondrial/oxidative phosphorylation dysfunction expression scores in skin compared with subgroup 1 and healthy controls. (**C**) Comparison of JDM skin expression signatures from tape stripping and full-thickness skin biopsies between the 2 identified subgroups. Unpaired 2-tailed parametric Student’s *t* test with Benjamini-Hochberg correction was used for the comparison between 2 groups. Cluster 1 RNA-Seq: *n* = 45 JDM_NL and 20 JDM_L samples corresponding to *n* = 28 patients; cluster 2 RNA-Seq: *n* = 10 JDM_NL and 2 JDM_L samples corresponding to *n* = 8 patients; cluster 1 microarrays: *n* = 6 CTL, 6 JDM_NL and 3 JDM_L samples/patients; cluster 2 microarrays: *n* = 0 JDM_NL and 6 JDM_L samples/patients.

To verify our findings from tape stripping expression data, we performed unsupervised hierarchical clustering of our previously published microarray data set from FFPE full-thickness skin biopsies of an independent JDM cohort (*n* = 15) (27) (Supplemental Figure 6). Within this independent JDM skin biopsy cohort, we also identified 2 JDM subgroups with distinct expression profiles, including *n* = 9 in biopsy subgroup 1 and *n* = 6 in biopsy subgroup 2 (Supplemental Figure 7). Similar to our JDM tape stripping expression dataset, we identified biopsy subgroup 2 as being characterized by dysregulation in the mitochondrial dysfunction pathway (Figure 4A) and a higher mitochondrial dysfunction signature score (Figure 4B). There were 2,575 DEGs in JDM biopsy subgroup 2 compared with subgroup 1 (FDR < 0.01, absolute log_2_ fold-change ≥ 1.0) (Figure 4A and Supplemental Table 2). A total of 1,160 of these genes were also regulated in the JDM tape stripping subgroup 2 compared with the JDM tape stripping subgroup 1, with 1,146 regulated in the same direction (all upregulated) and representing protein ubiquitination (*P* = 4.25 × 10^–20^), mitochondrial dysfunction (*P* = 2.68 × 10^–9^), and oxidative phosphorylation (*P* = 9.32 × 10^–11^) among other regulated pathways (Figure 4C, Supplemental Table 2, and Supplemental Table 3). Most of the lesional skin biopsy samples clustered in biopsy subgroup 2, with only 3 in subgroup 1. All NL skin biopsy samples clustered in biopsy subgroup 1. Three patients with JDM naive to systemic treatment at the time of biopsy clustered in biopsy subgroup 2 and only 1 in biopsy subgroup 1.

### Skin as compared with whole blood expression signature more effectively highlights a JDM disease endotype.

**Figure 5:**
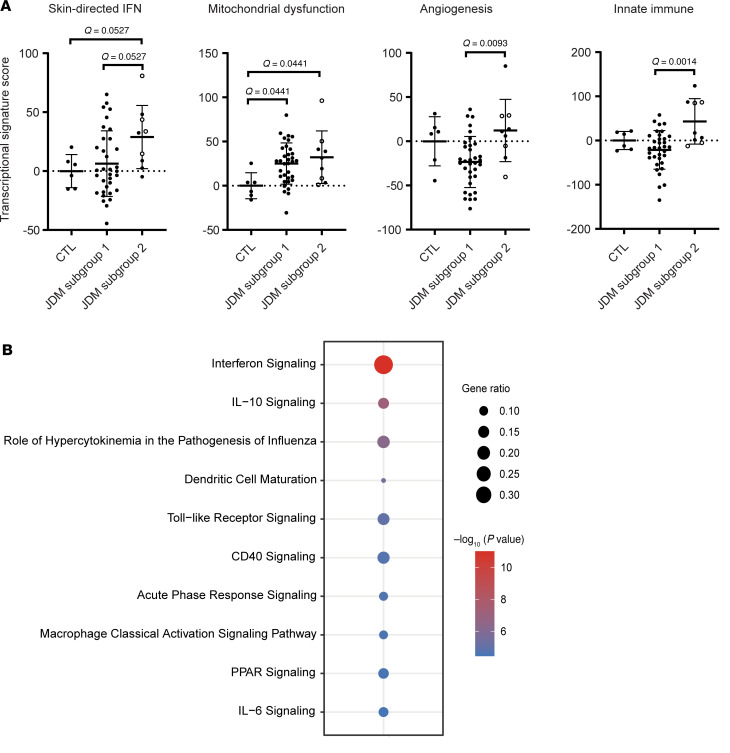
Whole blood as compared with skin transcriptional profile to define subgroups. (**A**) Blood expression scores for pathways of interest. Patients with both NL and L skin samples that were split between the 2 defined molecular subgroups are represented by unfilled circles. The blood samples were assigned to subgroup 2 for analysis. (**B**) Top 10 canonical pathways from the DEGs in L skin from JDM patients with lower Manual Muscle Testing scores (MMT-8 ≤ 146) compared with controls (FDR < 0.10). Unpaired 2-tailed parametric Student’s *t* test with Benjamini-Hochberg correction was used for the comparison between 2 groups. CTL: *n* = 6; cluster 1: *n* = 34 JDM samples; cluster 2: *n* = 9 JDM samples. Canonical pathway *P* values were computed by the IPA software.

As whole blood samples were also collected at the time of tape strip sampling if clinical labs were drawn, we evaluated if/how the expression scores from the pathways described above in the skin were reflected in the blood. Based on the 2 identified skin subgroups, we also observed higher skin-directed interferon, angiogenesis, and innate immune scores in the blood from skin subgroup 2 as compared with skin subgroup 1 and CTL (Figure 5A). The identification of a distinct biologic signature not only from skin but also from blood suggests that expression signatures from skin may have the potential to reflect systemic disease. Interestingly, while mitochondrial dysfunction scores from blood were higher in both of our JDM skin subgroups relative to CTL, they were not higher in skin subgroup 2 relative to subgroup 1 (Figure 5A, second panel), which suggests that the blood mitochondrial signature alone could not differentiate the subgroups (Supplemental Figure 7). Thus, the finding of a mitochondrial dysfunction expression signature identifying subgroup 2 was unique to skin (Figure 3, A and B). Similarly, the other pathway scores in blood did not highlight biological differences from the skin-derived subgroup 2 as effectively as seen in skin (Figure 5A compared with Figure 3B).

Upon independent unsupervised hierarchical clustering of blood expression data, we also identified 2 general clusters, but blood subgroups were not as well defined by a clear biological pattern or unique expression signature (Supplemental Figure 8). Patients from skin-derived subgroups were split between blood subgroups (Supplemental Figures 4 and 8), which suggests that transcriptomic skin signatures derived using tape stripping may be able to differentiate a unique patient subgroup, which is not identified in blood.

### Patients with JDM with higher muscle disease activity demonstrate increased interferon and myeloid-derived expression signatures in L skin.

We also performed differential expression analysis in both L tape stripping and blood expression datasets after stratifying patients with JDM by median organ-specific clinical disease activity scores for skin, muscle, and global disease activity (CDASI activity score of 7, MMT-8 score of 146, and PGA score of 3, defined by median of all patients in cohort). Interestingly, we identified the highest number of DEGs in the tape stripping expression dataset when comparing JDM disease activity subgroups with CTL after stratifying patients by degree of muscle disease activity (MMT-8 score ≤ 146, *n* = 1,198 DEGs with FDR < 0.10) as compared with skin (CDASI activity score ≥ 7, *n* = 20 DEGs with FDR < 0.10) or global disease activity (PGA score ≤ 3, *n* = 88 DEGs with FDR < 0.10). (Supplemental Table 2). The top upregulated pathways included interferon signaling (*P* = 1.0 × 10^–11^), IL10 signaling (*P* = 7.4 × 10^–8^), role of hypercytokinemia in the pathogenesis of influenza (*P* = 4.9 × 10^–7^), dendritic cell maturation (*P* = 2.1 × 10^–6^), Toll-like receptor signaling (*P* = 9.5 × 10^–6^), CD40 signaling (*P* = 1.8 × 10^–5^), and macrophage classical activation signaling pathway (*P* = 2.6 × 10^–5^) (Supplemental Table 3 and Figure 5B). In contrast, there were no DEGs noted in the blood expression dataset when the same groups of patients were compared with CTL, indicating the potential clinical utility and possible higher sensitivity of tissue-specific expression signatures in assessing disease activity.

### Tape stripping may capture immune cell signatures and suggests a predominant myeloid cell signature in JDM relative to CTL.

**Figure 6:**
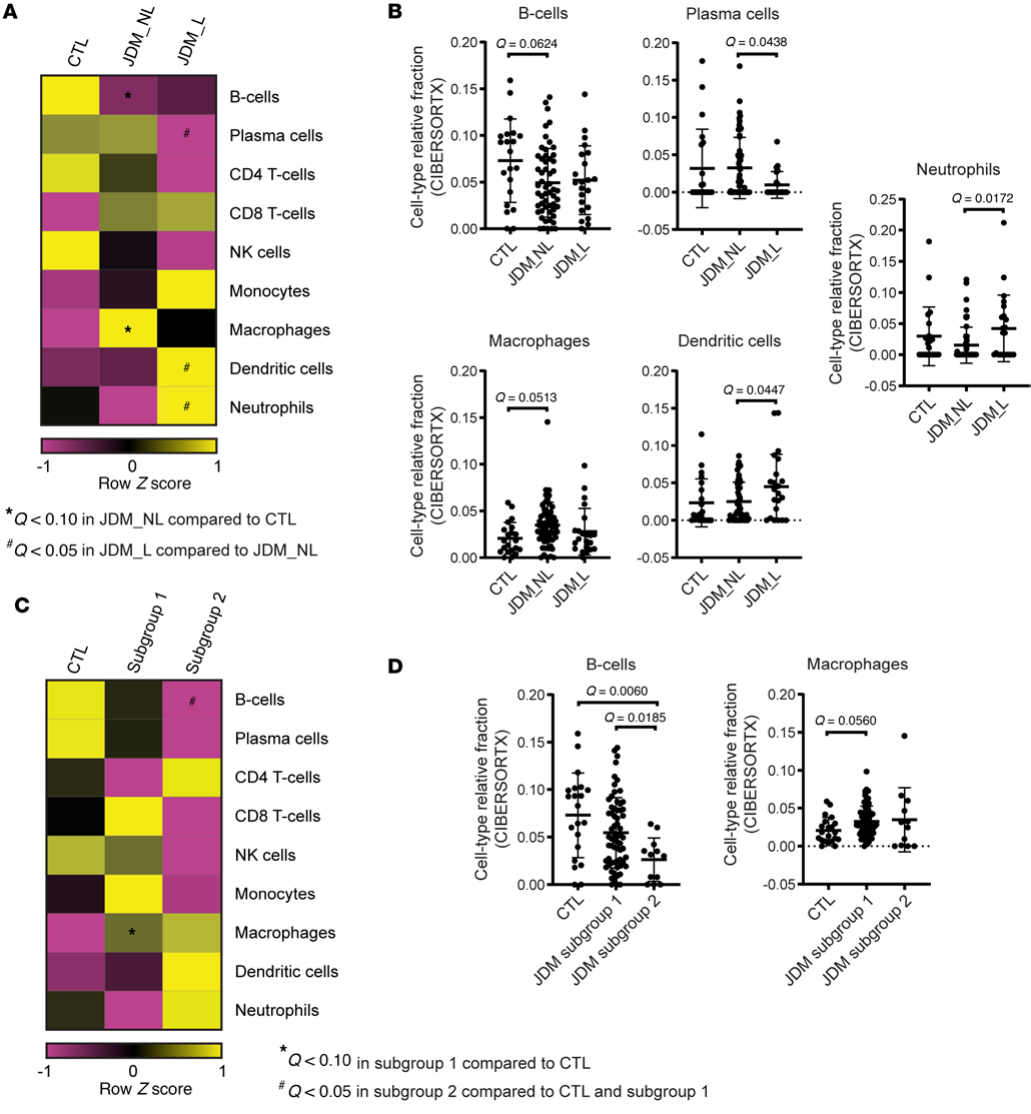
Immune cell enrichment analysis in JDM and control skin using CIBERSORTx. (**A**) Heatmap from each relevant immune cell type relative fraction in CTL (*n* = 21), NL (*n* = 55) JDM, and L (*n* = 22) JDM skin. (**B**) Graphs illustrating the relative fraction of B cells, plasma cells, macrophages, dendritic cells, and neutrophils as computed by CIBERSORTx in CTL and JDM samples (each dot represents 1 sample). (**C**) Heatmap from each relevant immune cell type relative fraction in each sample from control skin and samples in each identified skin subgroup. (**D**) Graphs illustrating the relative fraction of B cells and macrophages as computed by CIBERSORTx in each CTL and JDM subgroup sample (each dot represents 1 sample). CTL: *n* = 21, JDM_NL: *n* = 55, JDM_L: *n* = 22, JDM subgroup 1: *n* = 65, JDM subgroup 2: *n* = 12. Unpaired 2-tailed parametric Student’s *t* test with Benjamini-Hochberg correction was used for the comparison between 2 groups.

Using the CIBERSORTx immune cell enrichment analysis tool on our JDM tape stripping expression dataset, we noted enrichment for immune cell expression signatures (Supplemental Table 7). Transcriptomic signatures reflecting myeloid cell populations were particularly represented in L JDM skin (Figure 6A and Supplemental Figure 9A). Immune cell enrichment scores for dendritic cells and neutrophils were higher in L relative to NL JDM skin (Figure 6, A and B, and Supplemental Figure 9A). JDM NL skin reflected a predominant macrophage expression signature and lower B cell signature relative to healthy CTL patients (Figure 6, A and B, and Supplemental Figure 9A), concordant with the enrichment we noted for innate immune activation pathways even in NL skin. JDM NL as compared with L skin demonstrated more enrichment for plasma cells (Figure 6, A and B, and Supplemental Figure 9A).

Upon analysis of immune cell expression scores in JDM skin subgroups, a lower B cell score was found to differentiate JDM skin-derived subgroup 2 from JDM skin-derived subgroup 1 (Figure 6C, Supplemental Figure 9B, and Figure 6D), highlighting that there may be a contribution from differing immune cell–derived signatures and responses in unique JDM disease endotypes. In comparison with healthy CTL skin, JDM skin subgroup 1 reflected a higher macrophage score (Figure 6C, Supplemental Figure 9B, and Figure 6D).

## Discussion

In this study, we establish that tape stripping expression signatures can highlight important biological pathways in JDM disease pathogenesis, similar to full-thickness biopsies. We identify a common innate immune activation signature in both L and NL JDM skin, emphasizing an important role for innate immune dysregulation in JDM even in the absence of clinically apparent skin inflammation. Interestingly, we highlight the ability of tape stripping expression signatures to distinguish JDM molecular subgroups that may lend insight into current clinical disease classification systems. We report the finding of mitochondrial dysfunction in tape stripping expression signatures from a more treatment-refractory JDM subgroup and replicate this in an independent JDM skin biopsy cohort. We demonstrate that skin expression signatures may have the ability to better categorize JDM molecular subgroups as compared with blood, highlighting the need for more investigation into tissue-specific disease signatures in JDM to develop precision medicine approaches to care.

Our study highlights that JDM NL skin reflects more similarities to JDM L skin than healthy CTL skin, even without clinically apparent rash. This finding aligns with what has previously been reported in CLE (17), psoriasis (14, 22), and atopic dermatitis (13, 15). Indeed, the pathways that we identify, including those involved in mitochondrial biogenesis and calcium and nNOS signaling, may highlight early biological pathway dysregulation in development of JDM skin inflammation. Alternatively, these expression differences could be secondary to predominant epidermal skin sampling and alteration in epidermal thickness that make it harder to capture deeper immune signatures in NL skin.

By comparing transcriptomic signatures from L and NL skin, we identified pathways that may be critical in development of clinical inflammation. Our study indicates a common innate immune signature in L and NL JDM skin, with upregulation of the inflammasome pathway and *CASP1* on network analysis. A potential role for NLRP3 inflammasome dysregulation in disease pathogenesis has been suggested in idiopathic inflammatory myopathies and other skeletal muscle disorders, including inherited myopathies (28). In dermatomyositis, IL1B and IL18 protein levels are elevated in serum, and gene expression levels are also increased in muscle tissue (29, 30). An *IL18* gene signature has also been shown to molecularly distinguish DM from CLE skin lesions in the presence of otherwise similar histopathology, highlighting a potentially unique role for IL18 in DM pathogenesis (31). Increased expression of IL1A has been noted within muscle capillaries of DM patients with chronic disease and persistent weakness despite obvious clinical inflammation, suggesting potential contributory involvement of the inflammasome pathway in chronic disease (32). Furthermore, increased IL1 signaling has even been identified in PBMC expression signatures from patients with clinically inactive JDM (21).

We validated major biological signatures captured via tape stripping through comparison with an independent JDM skin biopsy cohort, suggesting that tape stripping is a feasible methodology for use in noninvasive assessment of pediatric autoimmune skin disease. While tape stripping may not capture deeper immune cell signatures, tape stripping has the potential to better assess keratinocyte-predominant expression signatures. In psoriasis, key proinflammatory genes, including *S100A12*, had more robust expression when obtained via tape stripping as compared with full-thickness biopsies (22). In our study, tape stripping signatures identified a common IFN signature in L skin and downregulation of mitochondrial biogenesis in NL skin. Tape stripping signatures also led to discovery of a JDM subgroup represented by mitochondrial dysfunction that we were then able to detect in our skin biopsy dataset. Overall, we noted more differences than similarities in tape stripping versus biopsy signatures, potentially reflecting predominant cell type origin due to differences in skin sampling depth, clinical cohort heterogeneity, and the transcriptomic platform utilized. Another study in atopic dermatitis skin that utilized tape stripping paired with either microarray or RNA-Seq profiling identified 217 common genes between techniques and highlighted that some genes were uniquely identified by RNA-Seq, including *TREM1* (15).

We identified a signature consistent with mitochondrial dysfunction in the skin of a subgroup of patients with JDM in our study. Although mitochondrial dysfunction has been reported in muscle and blood in JDM (33–35) and in 4 treatment-naive JDM skin biopsies (21), our study adds to this literature by reporting mitochondrial dysfunction using tape stripping methodology in both L and NL skin and within a specific JDM subgroup. Given that JDM patients with a cutaneous mitochondrial dysfunction signature were more treatment refractory, this suggests a potential role for mitochondrial dysfunction in the pathogenesis of a chronic or treatment-refractory disease course. Interestingly, the top upstream regulator of the mitochondrial dysfunction pathway in these patients was *NFE2L2*. NFE2L2 or Nrf2 is a major regulator of redox balance and influences mitochondrial bioenergetics, interferon signaling, and innate immune signaling (36, 37). NFE2L2-deficient mice have been shown to have reduced muscle force-generating capacity and impaired maximal activity and have downregulation of proteins associated with mitochondrial dysfunction (38, 39). If NFE2L2 influence on downstream targets contributes more specifically to disease pathogenesis in a subgroup of patients with JDM with chronic or treatment-refractory disease, it is possible that further investigation into NFE2L2 (and its downstream targets) could assist in personalized, pathway-based biomarker development and treatment targeting (40).

We noted in our study that molecular subgroups identified in skin also had a different biologic signature in blood, suggesting that skin subgroups may reflect broader systemic disease endotypes. JDM patients with more severe muscle disease also expressed a subset of DEGs in skin, reinforcing that skin signatures may reflect disease activity in other organs. This finding is supported by another recent study demonstrating that muscle and skin expression signatures have the most overlap in both up and downregulated DEGs as compared with blood (21). Within our study, skin as compared with blood subgroup signatures also better revealed underlying dysregulated biological pathway themes, indicating that tissue-specific expression signatures may aid in improved disease classification. Despite predominant epidermal sampling, we were still able to capture potential immune cell expression signatures in our tape stripping data using the CIBERSORTx analytical tool, and we identified a higher myeloid cell expression signature in JDM L and NL skin relative to healthy CTL. Our data are consistent with the predominant myeloid cell signature recently described in adult DM and in our independent JDM cohort using imaging mass cytometry (24, 25). In our dataset, JDM NL skin had the highest enrichment score for macrophages, reflecting the innate immune activation we identified on differential expression and pathway analysis in NL as well as L JDM skin. It is possible that immune cell signatures from tape stripping originated from dead or dying cells, extracellular vesicles, immune cell infiltrate into the epidermis of inflamed skin, or potentially sampling of deeper skin layers (18).

The results of our study represent findings from a single-center cohort, and the majority of patients had low disease activity while on treatment. It will be an important effort to study tape stripping expression signatures across multiple JDM cohorts to replicate results and to begin to better understand associations with clinical disease variables. Although RNA captured from cells via tape stripping methodology is of lower quantity and quality than standard skin biopsy, we were able to use a higher number of tape strips per sampling site and low-input RNA kits for cDNA library generation and deeper sequencing to make best use of these patient-oriented data. In the future, we plan to focus on the association of tape stripping signatures with treatment response in a newly diagnosed JDM cohort. We also hope to understand how JDM tape stripping signatures might highlight unique biological pathways in JDM pathophysiology as compared with other pediatric autoimmune diseases, such as lupus.

In conclusion, we demonstrated that noninvasive tape stripping captures important signatures reflective of underlying pathophysiology in JDM. In our dataset, tape stripping was able to identify possible molecular pathomechanisms underlying JDM disease endotypes, which were also reflected in blood. Skin-specific transcriptomic signatures may have the potential to lend insight into disease classification and precision medicine approaches to JDM care.

## Methods

### Sex as a biological variable.

Our study included both female and male patients (Table 1 and Supplemental Table 1); sex was included as a biological variable in the differential expression analysis.

### Study design and patient recruitment.

All patients with JDM (*n* = 28) and CTL patients (*n* = 20) were recruited from the pediatric rheumatology clinic at the University of Michigan C.S. Mott Children’s Hospital. All participants were between the ages of 18 months and 21 years at the time of recruitment (Table 1 and Supplemental Table 1). All patients with JDM had a confirmed diagnosis by a pediatric rheumatologist and met the 2017 EULAR/ACR classification criteria for JDM (1). Healthy CTL patients had no active rash, infection, or autoimmune or inflammatory disease. All JDM and CTL patients were enrolled into a cross-sectional cohort with onetime skin sampling, and JDM patients with treatment-naive or active disease were enrolled into a longitudinal cohort with additional skin sampling at clinical follow-up visits. A total of 16/28 (57.1%) of patients with JDM had at least 1 additional unique skin sample collected at a follow-up visit (total number of follow-up samples = 26 NL and 4 L). All procedures in this study were approved by the University of Michigan Internal Review Board.

### Clinical data collection.

Clinical data for patients with JDM were collected to align with the developed consensus core dataset for clinical use to inform research (41). Overall disease activity of patients with JDM was categorized by the treating physician at the time of study enrollment as (a) treatment naive (new diagnosis), (b) active disease (on medication), (c) flare, (d) inactive disease (on medication), or (e) inactive disease (off medication). Organ-specific disease activity assessments were also obtained for skin and muscle. Skin disease activity was assessed using the Cutaneous Dermatomyositis Disease Area and Severity Index (42, 43). Muscle disease activity was assessed by the Childhood Myositis Assessment Scale (44) and Manual Muscle Testing (45, 46) scores. Global disease activity was assessed by the PGA (47). Other organ involvement was assessed by the presence or absence of organ-specific features. MSAs were assessed for clinical care using the Myomarker Panel 3 (Mayo Clinic Laboratories). Detailed diagnostic characteristics and lab and immunosuppressive treatment variables were also collected (Table 1, Table 2, and Supplemental Table 1). All clinical data were stored electronically in a REDCap database.

### Biosample collection.

We collected tape stripping samples (both NL and L skin, if available) at each study visit and peripheral blood samples in BD PAXgene RNA if patients were having clinical labs drawn. Skin was sampled using 20 consecutive tape strips at standardized sampling sites. For NL skin, we sampled non-sun-exposed skin on the upper outer thigh for both JDM and CTL patients, and for L skin, we sampled Gottron’s papules over the metacarpophalangeal (MCP) joints if present for patients with JDM. If sampling of Gottron’s papules over MCP joints was not possible, we sampled Gottron’s sign or papules from an alternate extensor joint, such as elbows or knees. We used a D-Squame pressure instrument to uniformly apply pressure with skin sampling. Tape strips were immediately placed onto D-Squame storage cards, transported on ice to the lab, and stored at –80°. Following the manufacturer’s instructions, BD PAXgene RNA tubes were inverted 10 times and left at room temperature for 2 hours prior to storing at –80°.

### Tape strip processing and RNA isolation from biosamples.

For mRNA extraction from tape strips, we placed individual tape strips in Eppendorf tubes and added RNeasy Lysis (RLT) buffer containing 2% β-mercaptoethanol to the first tube, followed by 3 ultrasonication cycles (cycle: 30 seconds on, 30 seconds off) using the Bioruptor Pico sonication system in the cold room. The lysate from the first tube was transferred to the second tube, and sonication was repeated, continuing until we pooled lysate from all 20 tape strips. mRNA was subsequently isolated from the pooled tape strip lysate using the QIAGEN RNeasy Plus Mini Kit. On average, 0.56 ng/μL and a total of 11.2 ng of RNA was isolated per skin sample. In sequential thawing batches, mRNA was isolated from BD PAXgene RNA tubes using PAXgene blood RNA kits following the manufacturer’s instructions.

### Total RNA-Seq, RNA-Seq data processing, and differential expression analysis.

The SMARTer Stranded Total RNA-Seq kit v3 – Pico Input Mammalian was used to generate RNA-Seq libraries, and Illumina sequencing was performed by the University of Michigan Advanced Genomics Core using the NovaSeq platform. STAR alignment (version 2.5.2) was conducted, and gene quantification mapping to the human reference using GENCODE (version 29) annotations was performed using HTSeq (version 0.6.1). After correcting for batch effect with ComBat, Limma-Voom was used for sample normalization and to determine DEGs (FDR ≤ 10%) between JDM L, JDM NL, and CTL groups, controlling for sex. We additionally performed differential expression analysis after categorizing patients with JDM into higher versus lower disease activity groups using the cohort’s median clinical disease activity scores (median MMT-8 score of 146, median CDASI activity score of 7, median PGA score of 3; FDR < 0.10). Genes with Entrez Gene IDs were used in downstream analyses.

### Canonical pathways, literature-based network analyses, hierarchical clustering, and dot plot and heatmap generation.

QIAGEN IPA software was used (December 2021, 2022, 2023 winter releases; March 2023 spring release; and October 2023 fall release) to identify regulated canonical pathways from DEGs. Dot plots were created from the IPA output using an in-house R script. The customized script used for creation of the dot plots is included with other analysis code in GitHub and can be accessed at https://github.com/CutaneousBioinf/JDM_skin_RNAseq/tree/main Literature-based gene networks were generated using the Genomatix Pathway System (GePS) software installed on a local server. Unsupervised hierarchical clustering was performed using the locally installed Array Track HCA-PCA standalone package, with Euclidean distance and Ward’s linkage (48–50). DEGs between the subgroups identified by unsupervised clustering were extracted in the TIGR MultiExperiment Viewer application version 4.9.0 (unpaired analysis). Heatmaps were generated using the Morpheus visualization software from the Broad Institute (https://software.broadinstitute.org/morpheus), using the “transform values: subtract row mean, divide by row standard deviation” option.

### Cell type enrichment analysis.

The immune cell type enrichment tool CIBERSORTx (51, 52) was run using the LM22 signature to determine and compare the potential immune cell type abundance in all CTL and JDM samples.

### Calculation of signature scores.

Skin-directed IFN score was calculated by using the published algorithm (53) that we previously applied (54). Mitochondrial dysfunction, angiogenesis pathway, innate immune, and *NFE2L2* scores, as well as the 23-gene JDM L and 100-gene JDM NL signature scores, were calculated using this same algorithm. A list of 40 genes from the IPA mitochondrial dysfunction pathway and differentially regulated between the 2 identified JDM subgroups were used for the mitochondrial dysfunction score calculation (Supplemental Table 8). The angiogenesis and innate immune pathway gene signatures were extracted, respectively, from Panther and Reactome through the GePS software. A total of 87 and 84 of the 100 genes in each pathway, respectively, were expressed in our dataset and used for the pathway score calculation (Supplemental Table 8). A list of 71 genes downstream of *NFE2L2* and upregulated in the identified JDM group 2 compared with group 1 was used for generating the *NFE2L2* score (Supplemental Table 6).

### Statistics.

Data were graphed and Pearson’s correlation and statistics were performed using GraphPad Prism version 10. Data are presented as mean ± SD. For comparison between 2 groups, unpaired 2-tailed parametric Student’s *t* test was used with the Benjamini-Hochberg correction. *P* or *q* values less than 0.10 were considered statistically significant. For clarity, only the most relevant values were reported if significant.

### Study approval.

All procedures in this study were approved by the University of Michigan Internal Review Board, and written informed consent and assent were obtained prior to participation.

### Data availability.

Data are registered and deposited in NCBI dbGaP and can be accessed under the accession number phs003884.v1.p1. The analysis code and customized script used for creation of the dot plots can be accessed in GitHub at https://github.com/CutaneousBioinf/JDM_skin_RNAseq/tree/main (commit ID b359544). We have provided a Supporting Data Values file with all values used to generate manuscript figures for reference and use.

## Author contributions

JLT was involved in research study design, biosample collection and processing, data acquisition and analysis, and writing the manuscript. CCB contributed to data analysis, table and figure generation, and writing. SMHV and S Matossian contributed to biosample collection and data acquisition. MEM and CG were involved in biosample processing and data acquisition. MR, NS, JAM, and S Mohan contributed to biosample collection and data acquisition. JEG contributed to study design, methods development, and data analysis. LCT was involved in study design, methods development, and data processing and analysis. JMK contributed to study design, methods development, and data analysis.

## Supplementary Material

Supplemental data

Supplemental tables 1-8

## Figures and Tables

**Table 3:**
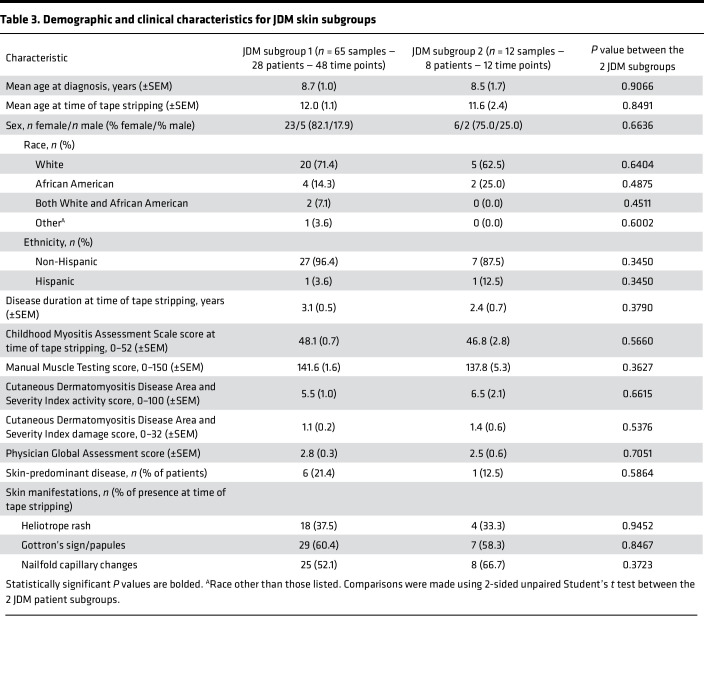
Demographic and clinical characteristics for JDM skin subgroups

**Table 2:**
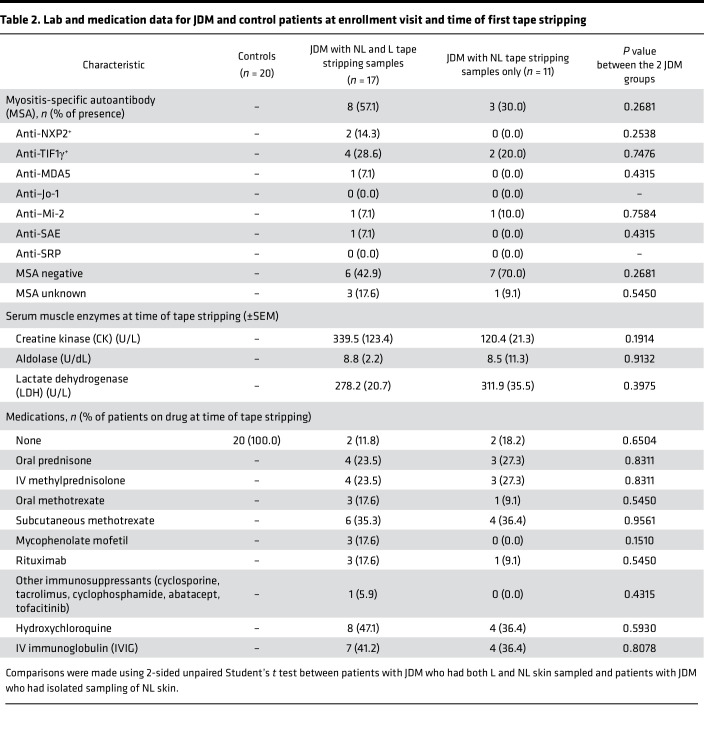
Lab and medication data for JDM and control patients at enrollment visit and time of first tape stripping

**Table 4:**
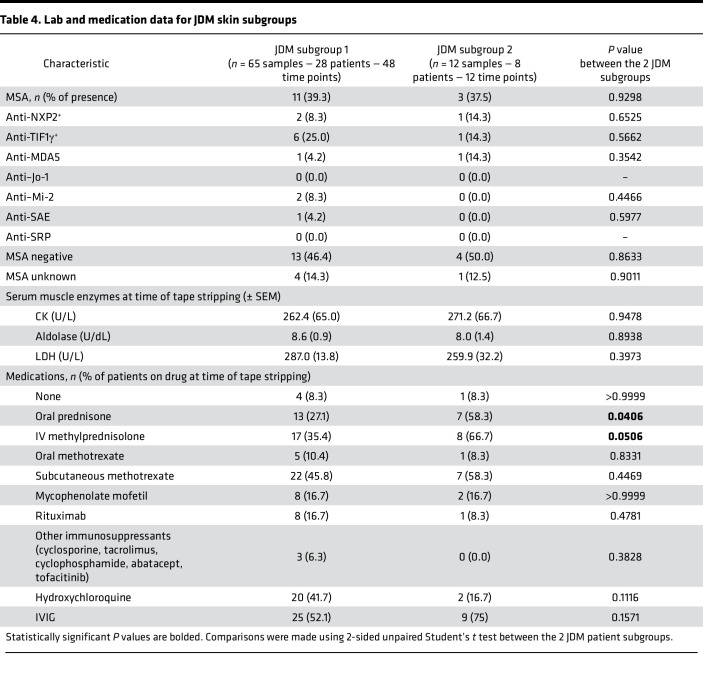
Lab and medication data for JDM skin subgroups

**Table 1:**
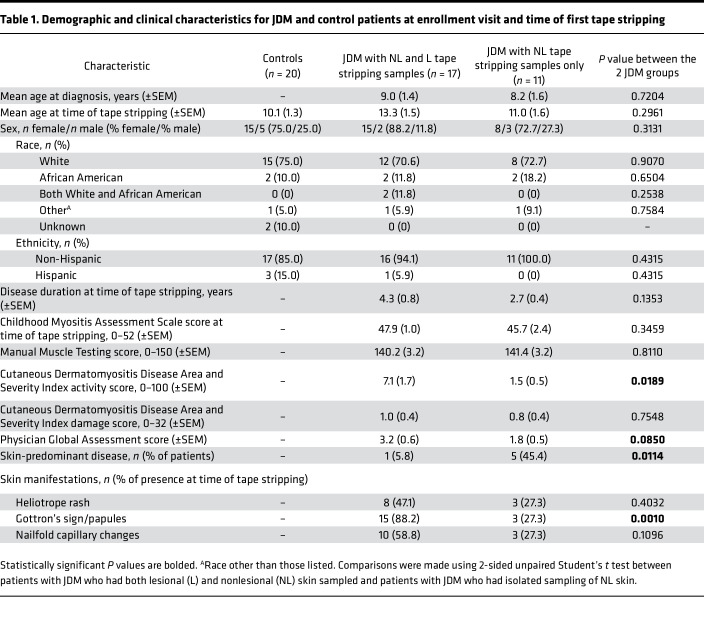
Demographic and clinical characteristics for JDM and control patients at enrollment visit and time of first tape stripping

## Version 1 03/13/2025

In-Press Preview

## Version 2 04/22/2025

Electronic publication
