# Supplementary material for: Tape strip expression profiling of juvenile dermatomyositis skin reveals mitochondrial dysfunction contributing to disease endotype
Source: JCI Insight. 2025 Mar 13;10(8):e179875. doi: 10.1172/jci.insight.179875 (PMC12016934; doi:10.1172/jci.insight.179875)
Supplement: Supplemental data [file jciinsight-10-179875-s232.pdf]

Supplemental Figure 1.

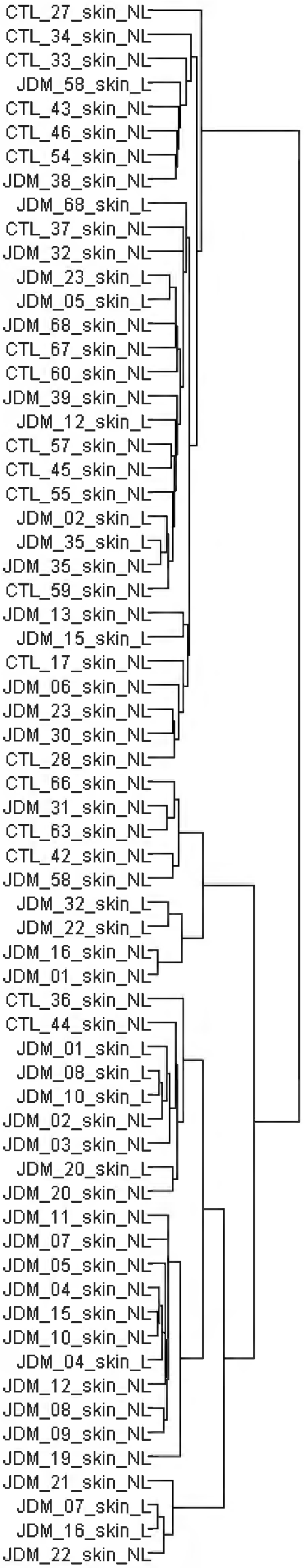

Supplemental Figure 2.

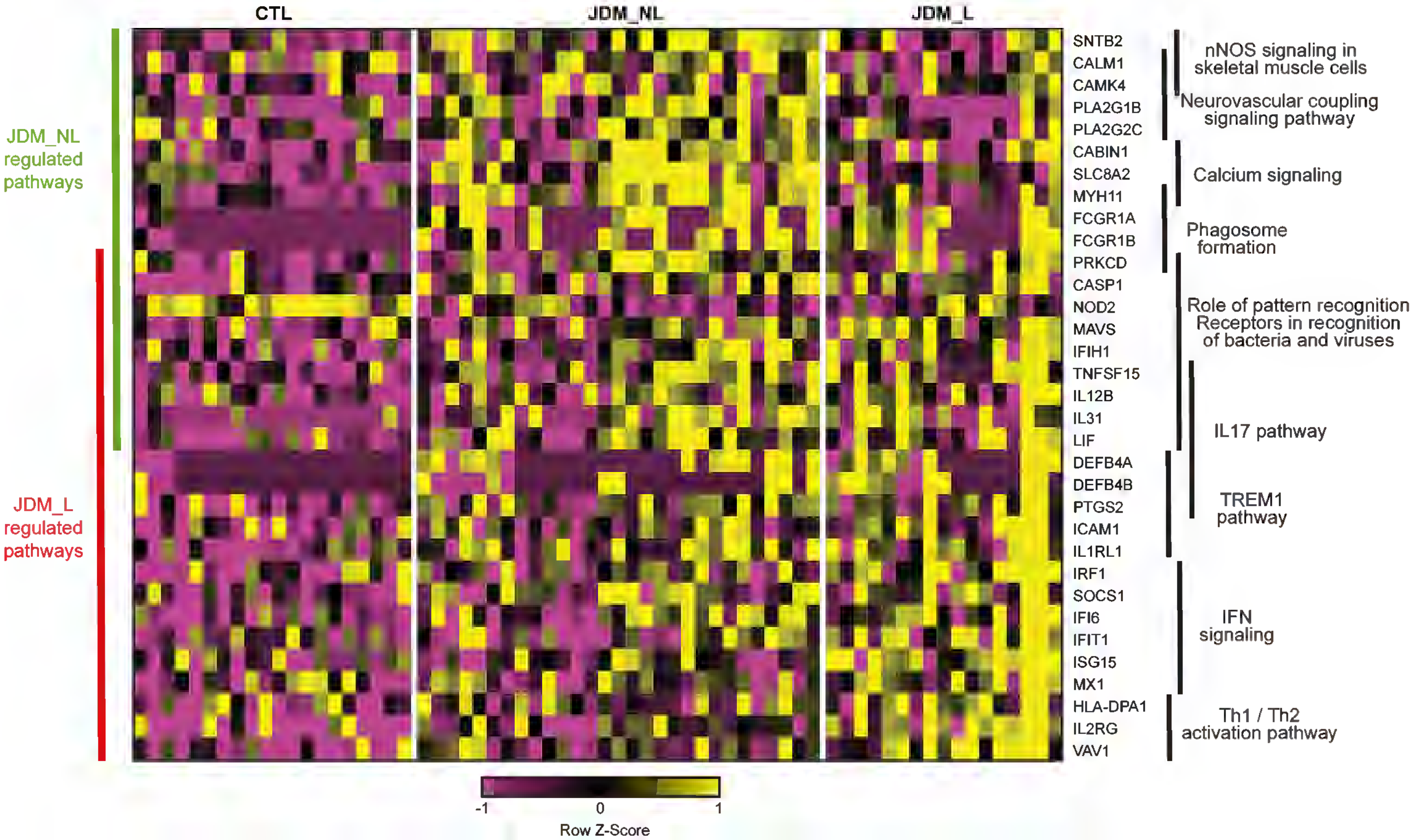

Supplemental Figure 3.

A.

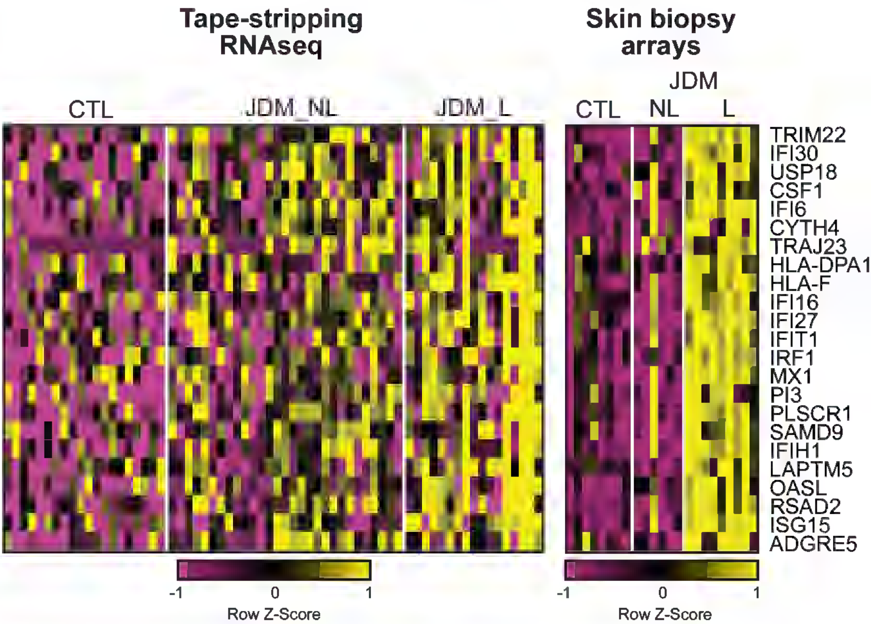

B.

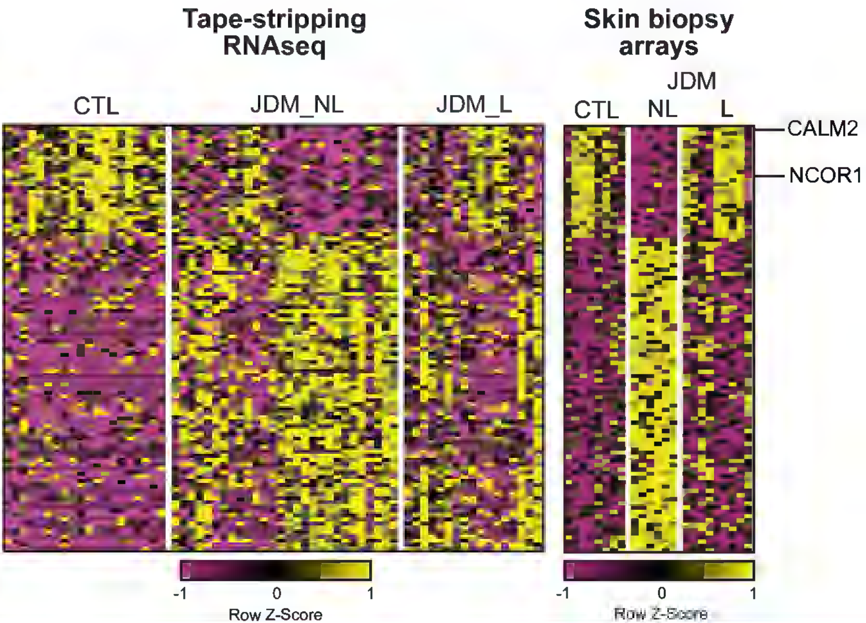

Supplemental Figure 4.

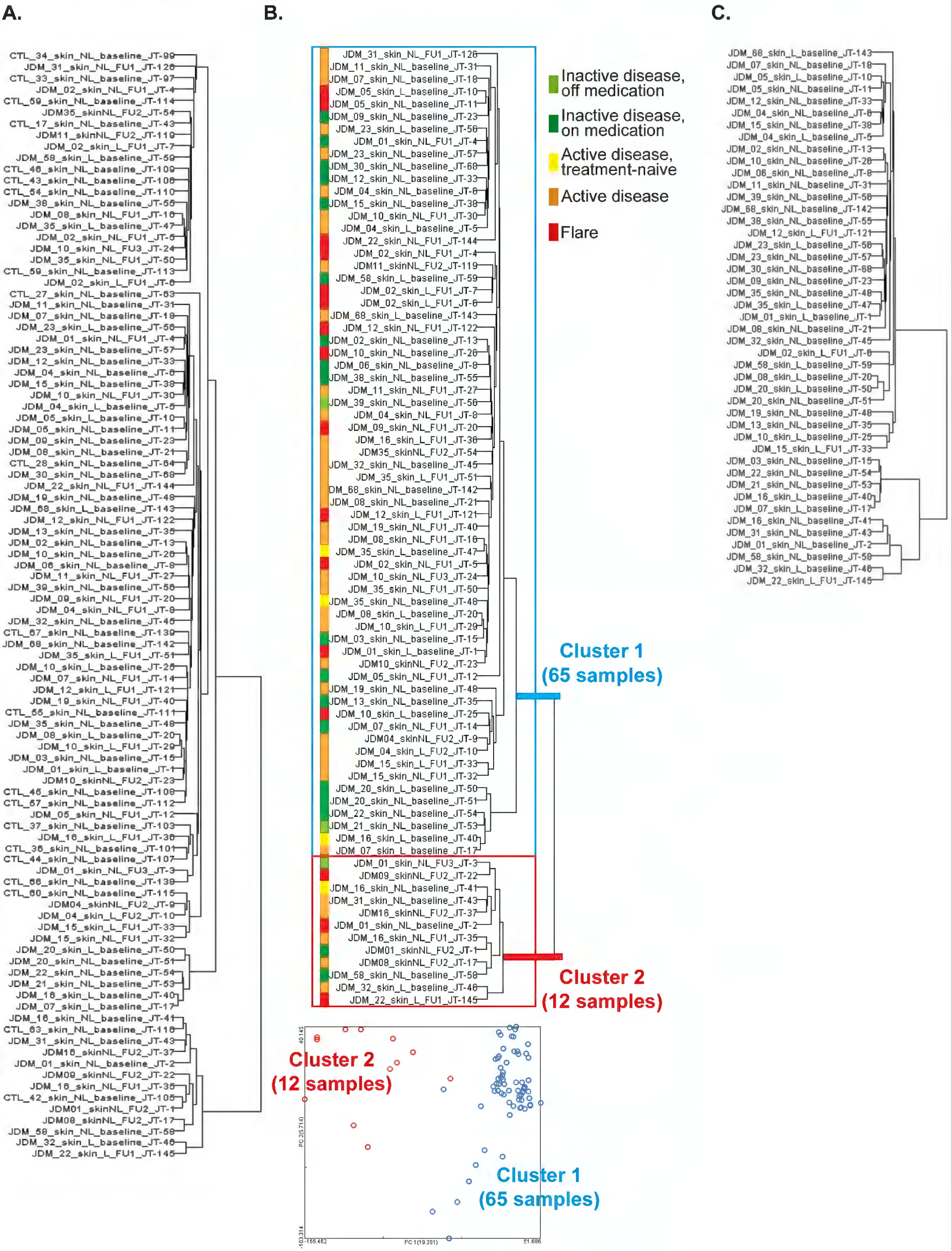

Supplemental Figure 5.

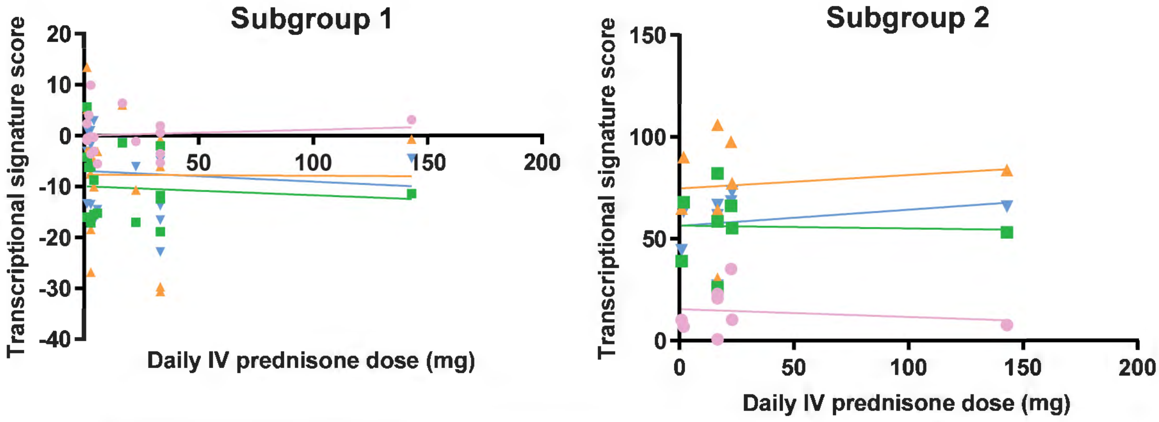

	Daily IV prednisone (mg)					
	Subgroup 1			Subgroup 2		
Transcriptional signature score	r	p-value	Number of XY Pairs	r	p-value	Number of XY Pairs
skin directed IFN score	0.0948	0.7174	17	-0.1585	0.7078	8
Mito_oxPho_score	-0.0860	0.7428	17	-0.0371	0.9305	8
Angiogenesis_score	-0.0060	0.9817	17	0.1285	0.7616	8
Innate Immune_score	-0.0897	0.7322	17	0.2400	0.5669	8

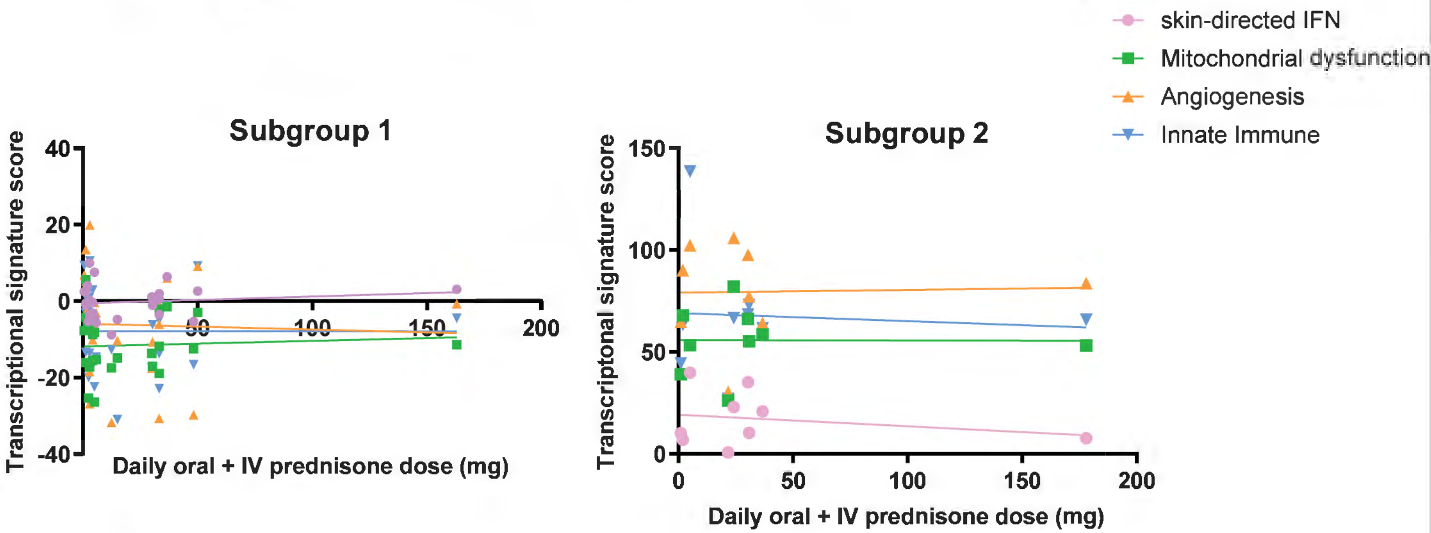

	Daily oral + IV prednisone (mg)					
	Subgroup 1			Subgroup 2		
Transcriptional signature score	r	p-value	Number of XY Pairs	r	p-value	Number of XY Pairs
skin-directed IFN	0.1339	0.5143	26	0.1339	0.5143	26
Mitochondrial dysfunction	0.0644	0.7546	26	-0.0074	0.9850	9
Angiogenesis	-0.0377	0.8550	26	0.0314	0.9361	9
Innate Immune_score	-0.0009	0.9966	26	-0.0713	0.8553	9

Supplemental Figure 6.

A.

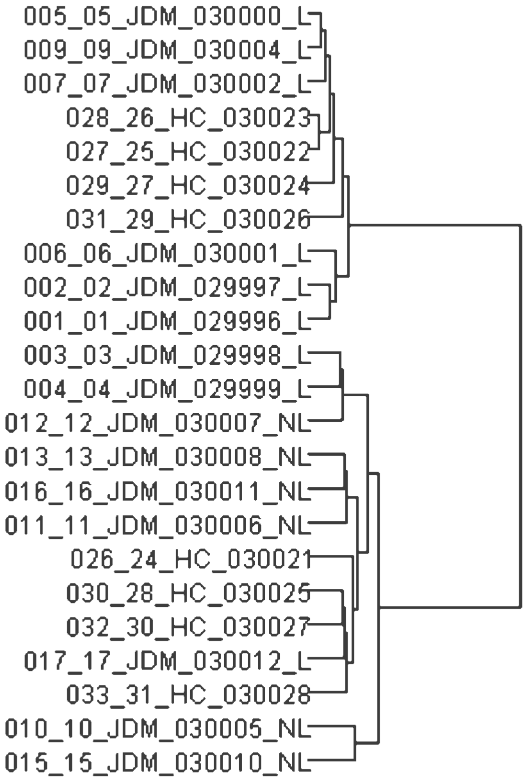

B.

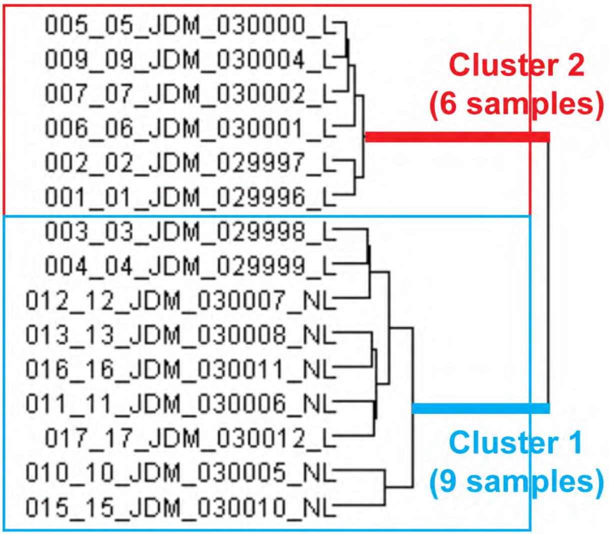

Supplemental Figure 7.

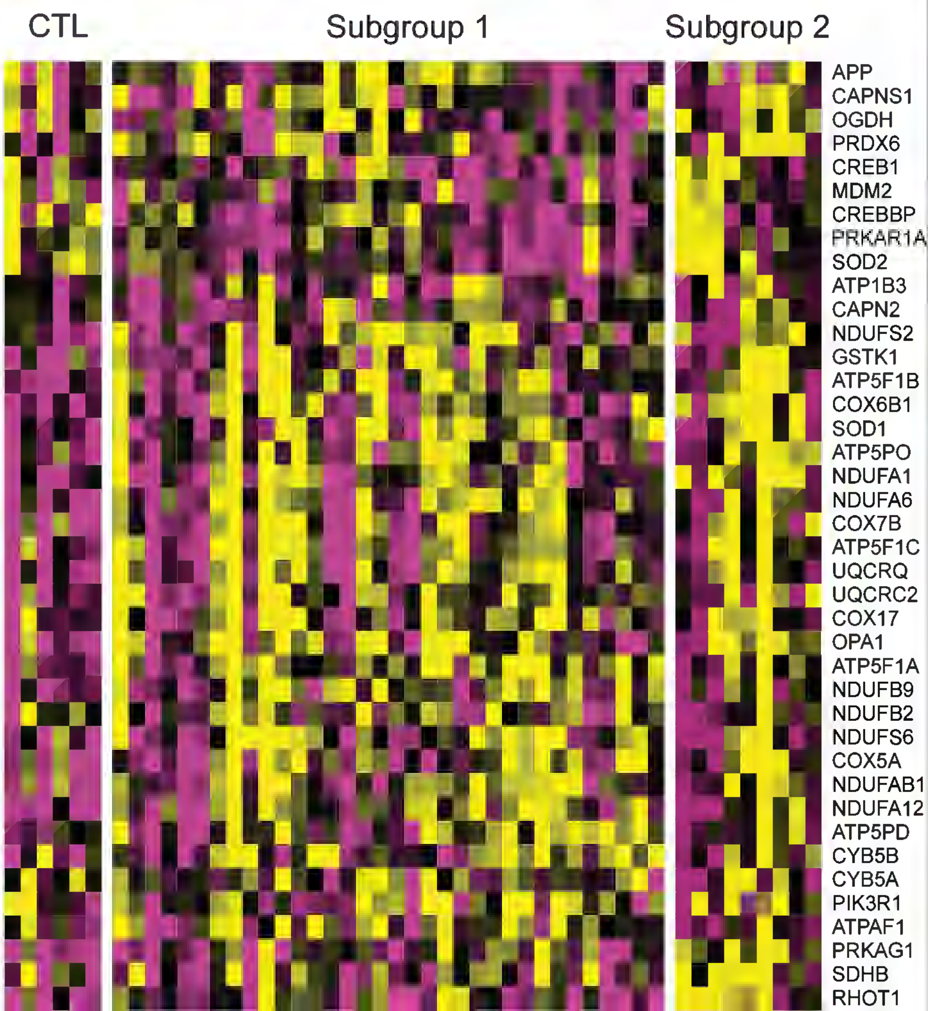

Supplemental Figure 8.

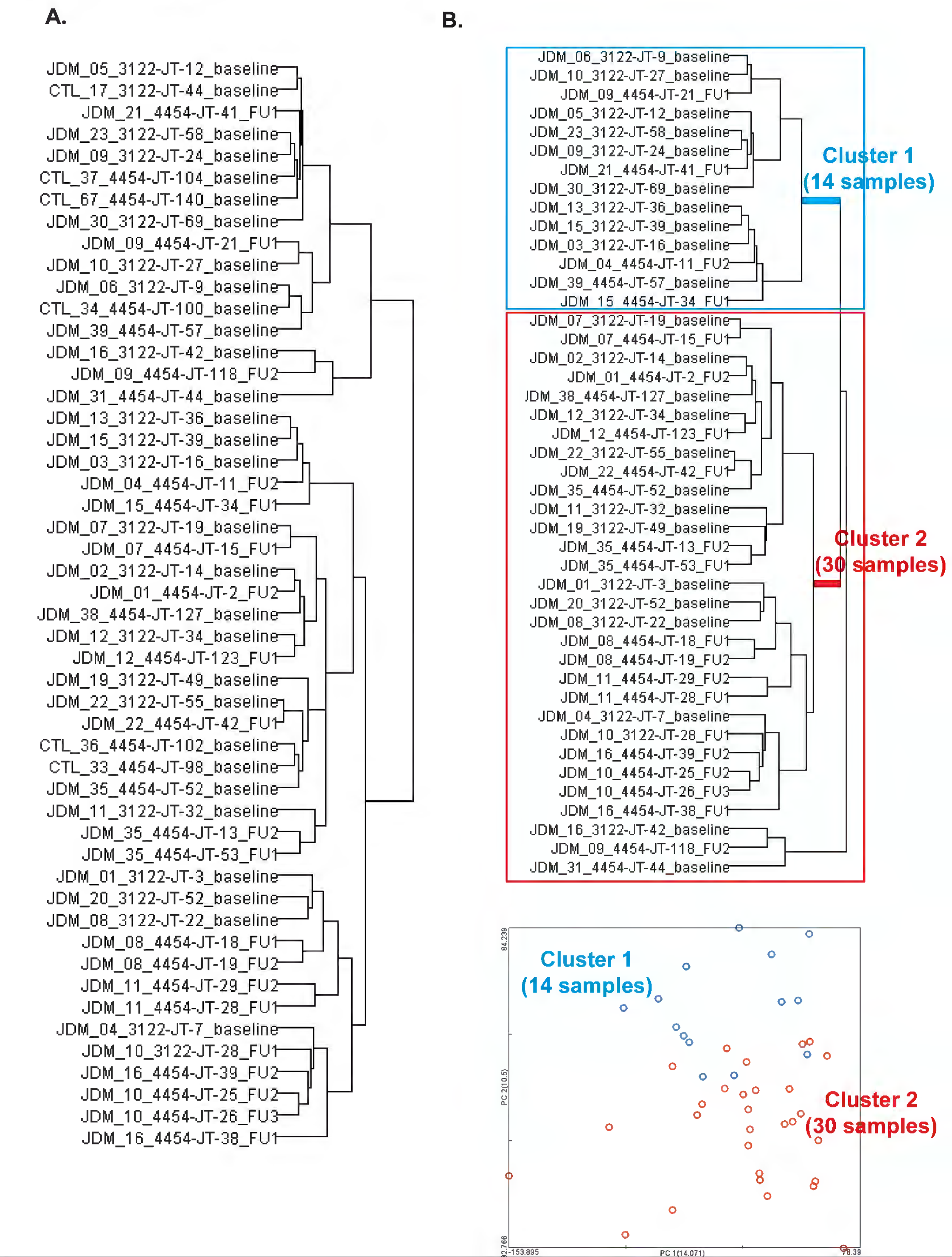

Supplemental Figure 9.

A.

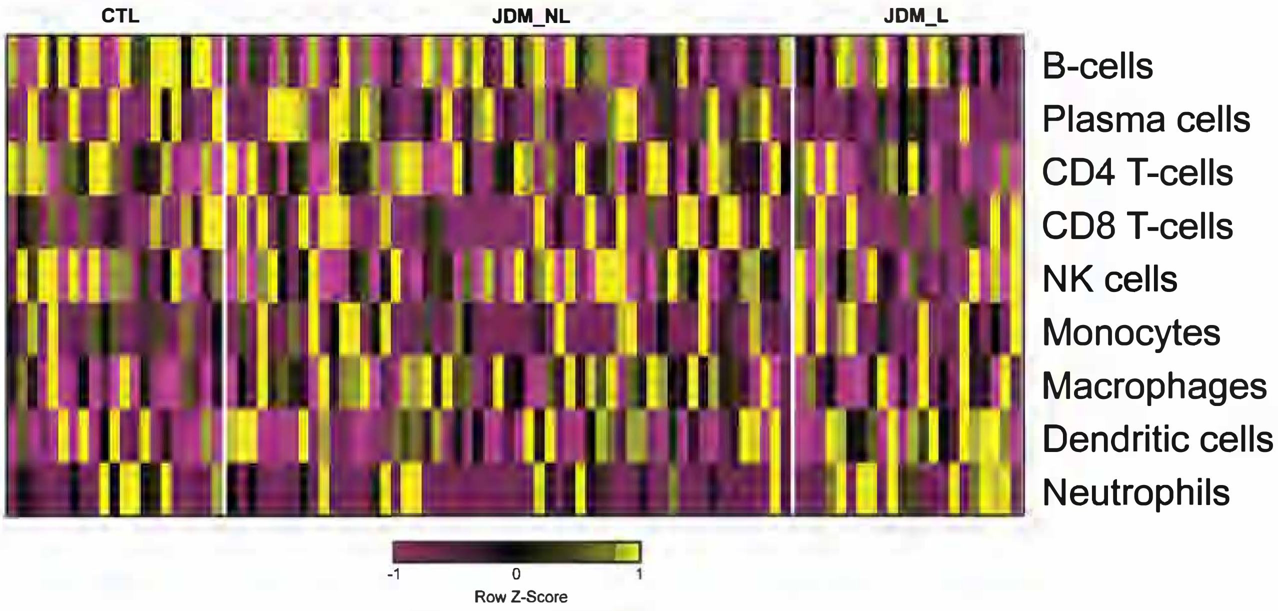

B.

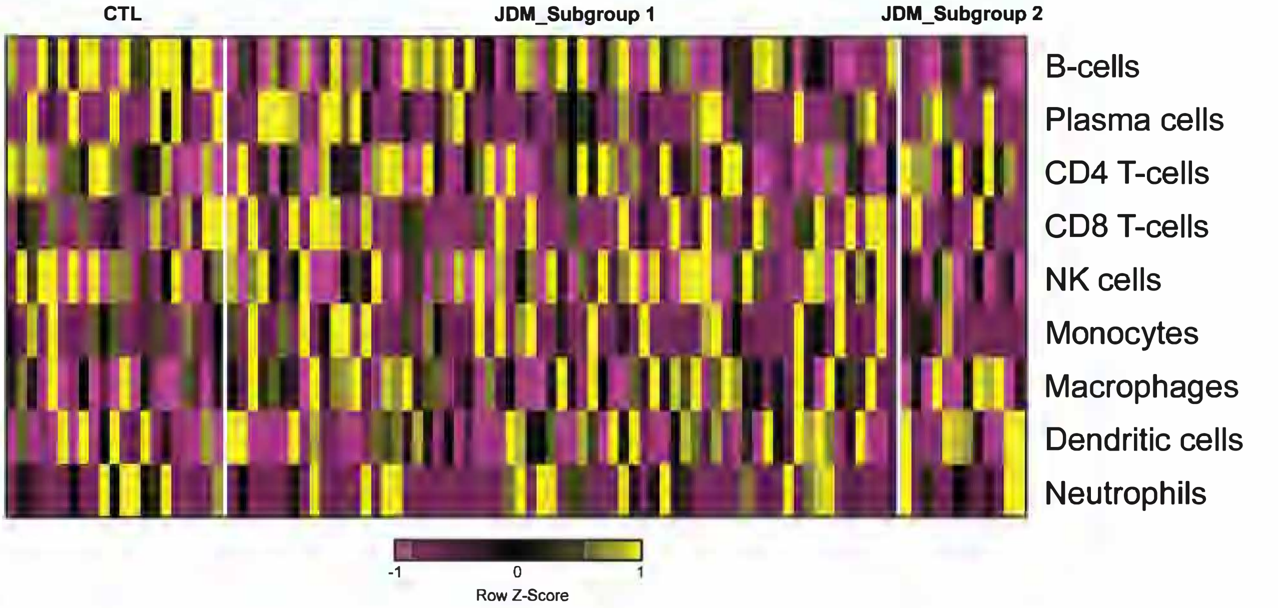

Supplemental material

Supplemental Tables

Supplemental Table 1. Full summary characteristics for JDM patients and controls at enrollment visit and time of first tape-stripping collection. Comparisons were made using two-sided unpaired Students' t-test between JDM patients who had both lesional (L) and non-lesional (NL) skin sampled and JDM patients who had isolated sampling of non-lesional skin.

Supplemental Table 2. Lists of differentially regulated genes analyzed in the manuscript.

Supplemental Table 3. IPA Canonical Pathway analyses.

Supplemental Table 4. IPA upstream regulator analyses.

Supplemental Table 5. Full clinical data for JDM skin subgroups. Comparisons were made using two-sided unpaired Students' t-test between the two JDM patient subgroups.

Supplemental Table 6. List of the 221 genes downstream of NFE2L2 from the JDM subgroup analysis.

Supplemental Table 7. CIBERSORTx enrichment analysis data.

Supplemental Table 8. Lists of genes used for transcriptional signature scores.

Supplemental Figures

Supplemental Figure 1. Unsupervised hierarchical clustering from tape-stripping expression data. From all control and JDM samples, combined time points.

Supplemental Figure 2 (detailed heatmap from Figure 1A). Biological signatures identified in JDM L and NL compared to control skin. Detailed heatmap of selected genes from relevant pathways.

Supplemental Figure 3 (detailed heatmaps from Figure 2). Comparison of tape stripping to full thickness skin biopsy expression signatures. A. 23-gene signature from overlap genes in lesional expression datasets. B. 100-gene signature from overlap genes in non-lesional expression datasets.

Supplemental Figure 4. Unsupervised hierarchical clustering from tape stripping expression data A. From all control and JDM samples, all time points. B. From all JDM samples only, all time points. C. From enrollment JDM samples only. Note that the 4 samples noted "FU1" are from patients who did not have a sample collected at the first visit, so those samples represent the enrollment samples.

Supplemental Figure 5. Correlation of steroid dose with skin-directed IFN, mitochondrial dysfunction, angiogenesis and innate immune transcriptional scores in the identified JDM skin sample subgroups. Pathway-based expression scores in skin also did not associate with steroid dose.

Supplemental Figure 6. Unsupervised hierarchical clustering from previous microarray full skin expression data. A. From all control and JDM samples. B. From all JDM samples only.

Supplemental Figure 7. Heatmap of 40 mitochondrial dysfunction genes from blood, using the skin-identified subgroups. While the expression of mitochondrial genes was significantly higher in subgroup compared to CTL and subgroup 2 compared to CTL, the two subgroups could not be distinguished.

Supplemental Figure 8. Unsupervised hierarchical clustering from blood expression data. A. From all control and JDM samples. B. From all JDM samples only.

Supplemental Figure 9 (detailed heatmaps from Figure 6). Immune cell enrichment analysis in JDM and control skin using CIBERSORTx. A. Heatmap from each relevant immune cell type relative fraction in control (CTL), non-lesional (NL) and lesional (L) JDM skin. B. Heatmap from each relevant immune cell type relative fraction in each sample from control skin and samples in each identified skin subgroup.
